# Valproate-Induced Hyperammonemic Encephalopathy Causing New-Onset Seizures

**DOI:** 10.7759/cureus.47288

**Published:** 2023-10-18

**Authors:** Aleena Sammar, Mena Tawfik, Fareha Fatima, Adam Butler, Kourtney Aylor-Lee

**Affiliations:** 1 Internal Medicine, Parkview Medical Center, Pueblo, USA; 2 Internal Medicine/Gastroenterology, Parkview Medical Center, Pueblo, USA

**Keywords:** phelan-mcdermid, seizure, valproic acid toxicity, urea cycle, valproate induced, hyperammonemia-encephalopathy, hyperammonemia, encephalopathy, valproic acid, valproate toxicity

## Abstract

Valproate-induced hyperammonemic encephalopathy (VHE) is a rare and severe side effect that can occur with valproic acid (VPA) therapy, despite therapeutic doses and normal serum levels of valproate. The typical signs of this condition include a sudden onset of impaired consciousness, focal neurologic symptoms, and an increase in seizure frequency. The exact cause of VHE is unknown, but it is believed to be related to the accumulation of toxic VPA metabolites and increased levels of ammonia that can cause swelling of the astrocytes and cerebral edema. We present a case of a 19-year-old male patient with a history of bipolar disorder on valproic acid 250 mg daily, admitted to the hospital after a new-onset seizure. He was found to have elevated levels of ammonia in his blood, despite having therapeutic levels of valproate and no liver dysfunction. His symptoms improved with discontinuation of the medication and his ammonia levels decreased. We discuss possible mechanisms and risk factors leading to encephalopathy while on valproate therapy. VHE should be considered a possibility when patients treated with valproate show signs of impaired consciousness.

## Introduction

Valproate is a branched-chain fatty acid that is widely used in therapy for epilepsy and psychiatric conditions such as bipolar and schizoaffective disorder [1,2]. It is generally considered safe, with a broad reference range of 50 to 100 mcg/ml; toxicity may occur at levels of 100-150 mcg/ml. However, there are serious side effects associated with valproate treatment including hepatotoxicity, coagulation disorders, pancreatitis, bone marrow suppression, and hyperammonemia [2]. One rare but severe side effect of treatment is valproate-induced hyperammonemic encephalopathy (VHE), which can occur even when liver function is normal and when valproate levels are within the therapeutic range. This condition can cause sudden onset of symptoms such as impaired consciousness, focal neurological deficits, and increased seizure frequency [1,3]. We report a patient who developed VHE and a new-onset seizure with normal valproate levels in the bloodstream. 

## Case presentation

A 19-year-old male with a past medical history of developmental delay caused by Phelan-McDermid syndrome, bipolar disorder on valproate therapy 250 mg at night, and autism spectrum disorder arrived at the hospital due to a new-onset seizure episode witnessed by his father. During the seizure episode, the patient's entire body jerked for 3 to 4 minutes, and he became confused afterward. However, he returned to his normal mental state after almost 15 minutes. Although he had never had seizures before, he regularly underwent electroencephalography due to his predisposition to them secondary to Phelan-McDermid syndrome. The previous electroencephalograms (EEGs) did not show any epileptiform discharges nor did the patient have a history of seizures in the past.

Upon arrival at the emergency room, the patient was afebrile, tachycardiac, normotensive, and saturating well on room air. The patient had a BMI of 26.85 kg/m^2^. He was alert and oriented at his baseline mentation. No asterixis or tremors were noticed on the physical exam. His lab results showed a leukocyte count of 16.7 x 10*9/L, an anion gap of 25 mmol/L, and CO_2_ of 15 mmol/L. Serum ammonia levels were 119 umol/L. Valproate levels were 21.5 ug/mL, and acetaminophen and salicylate levels were undetectable (Table 1).

**Table 1:** Lab values on presentation

Component	Value on presentation	Reference range and units
Sodium	143 mmol/L	136 – 145 mmol/L
Calcium	9.2 mg/dL	8.5- 10.1 mg/dL
Bicarbonate	15 mmol/L	20-31 mmol/L
Anion gap	25 mmol/L	6-14 mmol/L
Ammonia	119 umol/L	11-32umol/L
Valproic acid	21.5 ug/mL	50-100 ug/mL
Leukocyte count	16.7 x10*9/L	4-10x 10*9/L
Hemoglobin	16 g/dl	13.7- 17.7 g/dl

Creatinine kinase levels, lactic acid, and liver function tests were within the normal range. CT and MRI of the brain showed no acute intracranial abnormality, space-occupying lesion, or pathological enhancement. No evidence of infection was found in clinical evaluation and imaging studies. The poison control center was consulted, and they recommended checking valproic acid (VPA) levels again in six hours. The patient was admitted to the medical service for further management. Valproate was held, and the patient was started on polyethylene glycol for hyperammonemia. Repeat valproate levels came back at 14.3 ug/mL, below the normal range (Table 2).

**Table 2:** Lab values after admission and treatment

Component	Lab values after treatment	Reference range and units
Sodium	140 mmol/L	136 – 145 mmol/L
Calcium	8.9 mg/dL	8.5- 10.1 mg/dL
Bicarbonate	23 mmol/L	20-31 mmol/L
Anion gap	7 mmol/L	6-14 mmol/L
Ammonia	< 10 umol/L	11-32umol/L
Valproic acid	14.3 ug/mL	50-100 ug/mL
Leukocyte count	9.7 x10*9/L	4-10x 10*9/L
Hemoglobin	14 g/dl	13.7- 17.7 g/dl

No further seizure episodes were witnessed, and the EEG did not show any evidence of seizures while in the hospital. Neurology was consulted, and they recommended continuing the patient on levetiracetam 500 mg twice daily to prevent any further seizures. The patient's mentation remained at baseline while in the hospital and repeat ammonia levels were normal. Behavioral health recommended continuing levetiracetam and holding valproate on discharge. The patient did not have a seizure afterward. He was discharged with levetiracetam 500 mg tablets twice daily with recommendations to follow up with the patient's psychiatrist for clinical and behavioral evaluation as well as medication management. Follow-up EEG outpatient was completed which did not show any further evidence of seizures.

## Discussion

The occurrence of valproate-induced hyperammonemia varies, ranging from 16% to 100% [4]. Patients with VHE typically have normal liver enzyme levels, which suggests that a mechanism other than hepatic cell injury is at play [1]. The exact mechanism of VHE is uncertain, but it is related to the buildup of toxic VPA metabolites and increased ammonia levels [5]. Ammonia metabolism primarily occurs through the urea cycle, with ammonia being a byproduct of converting amino acids to α-ketoacids. In the liver, ammonia is transformed into urea, which is then excreted in the urine. VPA inhibits the activity of carbamoyl phosphate synthetase I, which is the first enzymatic reaction in the urea cycle, preventing the excretion of ammonia and raising plasma ammonia levels [6,7]. Hyperammonemia can cause encephalopathy by inhibiting glutamate uptake by astrocytes [5,6]. Ammonia-exposed astrocytes produce more glutamine, but its release is inhibited. The high level of glutamine increases intracellular osmolarity, leading to water influx, astrocytic swelling, and cerebral edema [4,5]. EEG is characterized by signs of severe encephalopathy with continuous generalized slowing, a predominance of theta and delta activity, occasional bursts of frontal intermittent rhythmic delta activity, and triphasic waves [6]. The risk factors for valproate-associated hyperammonemia include underlying urea cycle enzyme deficiencies, underlying liver disease, long-term valproate treatment, concomitant anti-epileptic drug therapy, particularly topiramate, strict vegetarianism, fasting, carnitine deficiency, and intellectual disability [1,2,8]. The most effective treatment is VPA discontinuation. Lactulose and levocarnitine are common treatments for VPA-induced hyperammonemia, with success rates of 41.8% and 50.0% [4]. Levocarnitine, the active isomer of carnitine, has been used to treat VHE resulting from a VPA overdose as well as regular dosages of VPA [4,5].

## Conclusions

It can be concluded that individuals undergoing valproate therapy may experience increased blood ammonia levels regardless of the duration of treatment. Patients who are undergoing long-term VPA therapy should be closely monitored for signs of hyperammonemia. It is recommended that patients who experience new neurological symptoms while on VPA treatment should have their blood ammonia levels checked, even if their VPA levels are within the normal range.
